# Correction: *Estimating health opportunity costs in low-income and middle-income countries: a novel approach and evidence from cross-country data*

**DOI:** 10.1136/bmjgh-2018-000964corr1

**Published:** 2019-06-04

Ochalek J, Lomas J, Claxton K. Estimating health opportunity costs in low-income and middle-income countries: a novel approach and evidence from cross-country data. *BMJ Global Health* 2018;3:e000964. doi: 10.1136/bmjgh-2018-000964

The license type for this paper has changed from CC BY-NC to CC BY.

